# Mitochondrial transcription factor A: linking mtDNA maintenance, mitochondrial stress responses, and inflammaging

**DOI:** 10.3389/fragi.2026.1830839

**Published:** 2026-06-24

**Authors:** Nengneng Shi, Xiaojing Li

**Affiliations:** 1 Health Science Center, Ningbo University, Ningbo, China; 2 Department of Gerontology, The Affiliated Hospital of Ningbo University, Ningbo, China

**Keywords:** age-related disease, aging, inflammation, mitochondria, mitochondrial transcription factor A, oxidative stress

## Abstract

Mitochondrial transcription factor A (TFAM) is a nuclear-encoded mitochondrial protein that directly binds mitochondrial DNA (mtDNA) and contributes to mitochondrial genome maintenance. Beyond its established roles in mitochondrial transcription, mtDNA packaging, nucleoid organization, replication support, and copy number control, TFAM is increasingly recognized as a potential regulator of aging-related mitochondrial stress responses. Because mtDNA instability, respiratory dysfunction, reactive oxygen species imbalance, impaired autophagy, cellular senescence, and chronic inflammation are closely interconnected during aging, TFAM may occupy a proximal position linking mitochondrial genome homeostasis to broader aging biology. However, TFAM should not be viewed as a uniformly protective factor. Its effects appear to depend on TFAM abundance, TFAM-to-mtDNA stoichiometry, tissue type, metabolic state, mitochondrial import, LONP1-mediated turnover, and mitochondrial quality-control capacity. TFAM deficiency may compromise mtDNA maintenance, impair oxidative phosphorylation, increase mitochondrial ROS production, and promote mtDNA-driven innate immune activation. Conversely, excessive or dysregulated TFAM accumulation may lead to mtDNA hypercompaction, reduce mtDNA accessibility, and potentially produce maladaptive effects in specific disease contexts. In this review, we discuss the structural basis of TFAM–mtDNA interaction, the role of TFAM in mtDNA transcription, copy number control, genome protection, damage handling, inflammatory signaling, cellular senescence, systemic aging, and age-related diseases. We also highlight therapeutic opportunities, limitations, and unresolved questions, emphasizing that future strategies should aim to restore TFAM homeostasis rather than simply increase TFAM expression.

## 1 Introduction

Aging is a progressive decline in physiological function and a complex biological process shaped by genetic, environmental, metabolic, and inflammatory factors. It is also a major risk factor for many chronic diseases. The updated hallmarks of aging include genomic instability, loss of proteostasis, disabled macroautophagy, deregulated nutrient sensing, mitochondrial dysfunction, cellular senescence, altered intercellular communication, chronic inflammation, and other interconnected processes (López-Otín et al., 2023). These hallmarks should not be interpreted as isolated events; rather, they form mutually reinforcing networks that drive tissue dysfunction over time.

Mitochondrial dysfunction is a central feature of aging. With advancing age, impaired oxidative phosphorylation, disrupted mitochondrial dynamics, accumulated mitochondrial DNA (mtDNA) damage, and weakened mitochondrial quality control can compromise cellular energy metabolism and promote the production of reactive oxygen species (ROS) (Adlimoghaddam, 2025). These abnormalities may further damage mtDNA and respiratory-chain components, thereby creating a self-amplifying cycle of mitochondrial stress. Another major feature of aging is chronic inflammation, commonly referred to as inflammaging. Aged tissues often exhibit persistent, low-grade sterile inflammation, which contributes to functional decline and increases susceptibility to age-related diseases (Li et al., 2023). Mitochondria are important contributors to this process not only through oxidative stress and ROS accumulation (Xu et al., 2025), but also through mtDNA-mediated innate immune activation. In particular, damaged or mislocalized mtDNA can function as a damage-associated molecular pattern (DAMP), thereby activating innate immune pathways and promoting sterile inflammation. In parallel, impaired macroautophagy may further accelerate aging by weakening the clearance of damaged organelles, misfolded proteins, and leaked nucleic acids. In aged cells, reduced autophagic flux can permit the accumulation of dysfunctional mitochondria and cytosolic mtDNA, thereby amplifying oxidative stress and innate immune activation (Zhong et al., 2022).

These aging hallmarks are closely related, at least in part, to defects in mitochondrial genome maintenance and mitochondrial quality control. Compared with upstream regulators such as sirtuin 1 (SIRT1), AMP-activated protein kinase (AMPK), and peroxisome proliferator-activated receptor γ coactivator 1α (PGC-1α), which mainly influence mitochondrial function through broader nuclear transcriptional and metabolic programs, TFAM acts as a downstream effector of these pathways and operates more directly at the level of mtDNA homeostasis. TFAM is not only involved in mitochondrial transcriptional regulation but also serves as a major mtDNA-binding protein that participates in mitochondrial nucleoid organization, mtDNA packaging, replication support, mtDNA copy number maintenance, and genome stability (Campbell et al., 2012; Kozhukhar and Alexeyev, 2023). On this basis, TFAM may function as a relatively downstream regulatory node linking upstream aging-associated stress pathways with downstream mitochondrial dysfunction, mtDNA instability, and inflammatory activation. Meanwhile, emerging evidence suggests that TFAM may also participate in inflammatory signaling pathways mediated by cytosolic mtDNA. Importantly, however, TFAM should not be viewed as a uniformly protective factor. Its effects are likely bidirectional and context dependent. Appropriate TFAM expression and activity may help stabilize mtDNA and support mitochondrial homeostasis, whereas abnormal TFAM accumulation or a disrupted TFAM-to-mtDNA ratio may aggravate mitochondrial dysfunction in specific cell types, stress states, or disease contexts.

This review summarizes the potential roles of TFAM in aging-related mitochondrial homeostatic imbalance. We focus on how TFAM may affect aging-related phenotypes through mitochondrial functional regulation, oxidative stress, mtDNA-driven inflammation, and macroautophagy. We also discuss the therapeutic potential and limitations of targeting TFAM in aging and age-related diseases, with emphasis on restoring TFAM homeostasis rather than indiscriminately increasing TFAM abundance.

## 2 Structural basis of TFAM-mtDNA interaction

Human mtDNA is a 16,569-bp double-stranded circular genome located in the mitochondrial matrix. It encodes 37 genes, including 13 essential oxidative phosphorylation polypeptides, 22 transfer RNAs, and 2 ribosomal RNAs (Yan et al., 2019). Unlike nuclear DNA, mtDNA is not wrapped by histones. Instead, it is organized into compact DNA-protein structures termed mitochondrial nucleoids. Among nucleoid-associated proteins, TFAM is one of the most abundant and functionally important structural components. TFAM is a nuclear-encoded basic protein of approximately 25 kDa that is synthesized in the cytoplasm and imported into the mitochondrial matrix. The newly synthesized TFAM precursor contains an N-terminal mitochondrial targeting sequence (MTS), which is proteolytically cleaved after mitochondrial import to generate mature TFAM in the mitochondrial matrix (Choi and Garcia-Diaz, 2022). Structurally, TFAM belongs to the high-mobility group B (HMGB) protein family and contains two HMG-box domains, HMG1 and HMG2, connected by a linker region and followed by a C-terminal tail (Gangelhoff et al., 2009). These domains cooperate to determine how TFAM recognizes, bends, packages, and compacts mtDNA.

TFAM interacts with mtDNA through both sequence-specific and sequence-independent binding modes. Sequence-specific binding occurs mainly at upstream recognition sites in mitochondrial promoter regions, including the light-strand promoter (LSP) and heavy-strand promoter (HSP). In this setting, TFAM binds upstream of the transcription start site, contacts the DNA through its HMG-box domains, and induces a sharp U-turn in the DNA. This structural distortion facilitates the recruitment and positioning of mitochondrial RNA polymerase and other transcriptional components (Ngo et al., 2014; Hillen et al., 2017; Rubio-Cosials et al., 2018). Thus, promoter-specific TFAM binding provides a structural basis for TFAM-dependent mitochondrial transcription initiation.

TFAM also binds mtDNA non-specifically across the mitochondrial genome. This binding mode is essential for mtDNA packaging and nucleoid formation. The structural consequences of TFAM binding depend strongly on the stoichiometric relationship between TFAM and mtDNA. Excess TFAM relative to mtDNA may lead to nucleoid hypercompaction and reduce the accessibility to the mitochondrial transcriptional machinery.

## 3 TFAM in mtDNA transcription, replication, copy number control, and damage handling

Mitochondrial DNA homeostasis depends on the coordinated regulation of mtDNA transcription, replication, copy number maintenance, nucleoid organization, and genome protection. This section summarizes how TFAM contributes to these processes and helps maintain mitochondrial genome stability.

### 3.1 TFAM-dependent mitochondrial transcription initiation

Mitochondrial DNA transcription is catalyzed by mitochondrial RNA polymerase (POLRMT), but efficient promoter-specific initiation requires the cooperation of TFAM and mitochondrial transcription factor B2 (TFB2M). TFAM binds upstream of the transcription start sites at LSP and HSP, induces a DNA U-turn, and helps recruit and position POLRMT at mitochondrial promoters. TFB2M is then incorporated into the promoter complex and promotes promoter melting, allowing POLRMT to initiate RNA synthesis (Malarkey et al., 2012; Bestwick and Shadel, 2013; Hillen et al., 2017; Falkenberg et al., 2024). After initiation, POLRMT dissociates from TFAM and TFB2M and enters the elongation phase with assistance from mitochondrial transcription elongation factor (TEFM). Experimental evidence suggests that transient TFAM overexpression can stimulate mitochondrial transcription in cultured cells, although it does not always cause a proportional increase in mtDNA copy number (Maniura-Weber, 2004).

### 3.2 TFAM and mtDNA copy number

Mitochondrial DNA exists as a multicopy genome, and its copy number differs substantially among cell types and tissues according to developmental stage, energy demand, and mitochondrial content. Most somatic cells contain hundreds to thousands of mtDNA copies, whereas oocytes contain much higher copy numbers (Van Blerkom, 2008; O’Hara et al., 2019). Because mtDNA encodes key components of the oxidative phosphorylation system, maintaining an adequate mtDNA copy number is important for mitochondrial respiratory capacity.

In addition to its role in generating mitochondrial mRNA, POLRMT also synthesizes the RNA primers required for the initiation of mitochondrial DNA replication (Young and Copeland, 2016). Subsequent mitochondrial DNA replication is accomplished by DNA polymerase γ (Pol γ), and the RNA primers are excised by proteins including ribonuclease H1 (RNase H1) and DNA replication helicase/nuclease 2 (DNA2) (El-Hattab et al., 2017). Consequently, TFAM plays a critical role in maintaining mtDNA copy number. Experimental studies show that TFAM depletion reduces mtDNA copy number, whereas TFAM overexpression can increase mtDNA copy number in several cellular and animal models (Seidel-Rogol, 2002; Kanki et al., 2004; Matsushima et al., 2010; Thomas et al., 2012). Conversely, mtDNA depletion also lowers TFAM protein levels, suggesting that mtDNA binding stabilizes TFAM and protects it from degradation (Seidel-Rogol, 2002). Thus, TFAM helps maintain mtDNA abundance, while mtDNA binding stabilizes TFAM.

Unbound or excess TFAM is more susceptible to LONP1-mediated degradation, whereas mtDNA-bound TFAM is relatively stable. Thus, inhibition of LONP1 may increase the TFAM-to-mtDNA ratio, although this does not necessarily indicate improved mitochondrial function. When TFAM accumulates excessively relative to mtDNA, mitochondrial transcription may be suppressed, possibly because densely coated mtDNA becomes less accessible to the transcriptional machinery (Matsushima et al., 2010).

Changes in mtDNA copy number are associated with aging and age-related diseases. Peripheral blood mtDNA copy number declines with age and has been associated with impaired cognitive function, reduced muscle strength, and increased mortality risk in older adults (Mengel-From et al., 2014). Reduced mtDNA copy number and respiratory-chain deficiency have also been reported in tissues from Alzheimer’s disease, Parkinson’s disease, and coronary heart disease (Coskun et al., 2004; Grünewald et al., 2016; Liu et al., 2017). In experimental models, TFAM overexpression can attenuate mtDNA copy number loss, improve respiratory-chain activity, and alleviate pathological phenotypes in models of myocardial infarction, heart failure, diabetic neuropathy, and brain aging (Ikeuchi et al., 2005; Hayashi et al., 2008; Thomas et al., 2012; Chandrasekaran et al., 2015; Ikeda et al., 2015).

Nevertheless, mtDNA copy number should not be used as a simple surrogate for mitochondrial quality. Its functional meaning depends on tissue type, mtDNA mutation burden, heteroplasmy, respiratory-chain integrity, mitochondrial turnover, and cellular metabolic demand (Filograna et al., 2021). In some contexts, increased mtDNA copy number may represent a compensatory response to mitochondrial stress rather than true functional improvement. Therefore, the relationship among TFAM, mtDNA copy number, and aging-related mitochondrial dysfunction should be interpreted in a tissue- and context-specific manner. Moreover, direct evidence conclusively linking TFAM, mtDNA copy number dynamics, and the aging process remains limited, and the mechanistic connections among these factors warrant further investigation.

### 3.3 TFAM in nucleoid organization

In addition to its roles in transcription and copy number control, TFAM is a major organizer of mitochondrial nucleoids. Under physiological conditions, TFAM coats mtDNA and contributes to the formation of compact nucleoprotein structures that preserve mtDNA stability.

The packaging and compaction mechanism of mtDNA has not been fully elucidated. However, several existing studies suggest that following non-specific binding to mtDNA, TFAM can induce the melting of several base pairs of mtDNA, increase the flexibility of mtDNA strands, and mediate mtDNA compaction via a flexible hinge-like mechanism (Farge et al., 2012; Traverso et al., 2015). TFAM can oligomerize and dimerize via antiparallel paired helices in its HMG-box domain (Kaufman et al., 2007; Ngo et al., 2011). Accumulating evidence demonstrates that non-specific binding of TFAM to mtDNA can induce U-turn formation, and both this effect and TFAM dimerization are indispensable for the efficient compaction of mtDNA (Kaufman et al., 2007; Rubio-Cosials et al., 2018). Studies have also shown that TFAM can induce cross-strand binding, which contributes to mitochondrial nucleoid assembly (Kukat et al., 2015). MtDNA is situated near the ROS-generating sites of the inner mitochondrial membrane (IMM), rendering it more susceptible to oxidative damage than nuclear DNA. By packaging and enveloping mtDNA, TFAM functions analogously to histones, physically shielding mtDNA from oxidative damage.

### 3.4 TFAM in mtDNA stability

Because mtDNA is located near respiratory-chain-derived ROS and lacks nuclear chromatin-like packaging, the mitochondrial genome is particularly vulnerable to damage. Oxidative lesions, abasic sites, strand breaks, and stalled replication intermediates can impair mitochondrial gene expression and contribute to mitochondrial dysfunction. TFAM participates in mtDNA damage handling, but its role appears to be dual and context dependent.

TFAM preferentially binds oxidatively damaged DNA, including 8-oxoguanine-containing mtDNA, which may stabilize damaged regions or contribute to lesion recognition (Yoshida et al., 2002). By coating mtDNA, TFAM may also reduce exposure of the mitochondrial genome to ROS and nuclease attack. These observations support a protective role for TFAM in maintaining mitochondrial genome integrity.

At the same time, dense TFAM binding may interfere with the access of base excision repair (BER) enzymes to damaged mtDNA. *In vitro* studies have shown that TFAM can compete with BER proteins for mtDNA binding sites, suggesting that excessive or tightly bound TFAM may slow mtDNA repair under certain conditions (Canugovi et al., 2010). Consistent with the need for an appropriate level of TFAM, TFAM knockdown has also been associated with increased accumulation of mtDNA damage (Canugovi et al., 2010), indicating that insufficient TFAM may compromise mtDNA protection and maintenance. Conversely, TFAM may also participate in the processing or clearance of severely damaged mtDNA. For example, TFAM can form covalent crosslinks with apurinic/apyrimidinic sites in mtDNA through lysine residues, thereby promoting DNA strand cleavage (Xu et al., 2019). Therefore, rather than acting simply as a BER inhibitor, TFAM may function as a context-dependent regulator of mtDNA damage triage. Dense TFAM coating may limit enzymatic access for repair, whereas TFAM binding to oxidized or abasic lesions may help mark damaged mtDNA molecules for elimination (Xu et al., 2019), thereby limiting the accumulation of unrepaired or abortive repair intermediates, the persistence or propagation of damaged genomes, and secondary mitochondrial stress. This interpretation may reconcile the apparently opposing observations that TFAM can restrict repair protein binding under some conditions while also contributing to mtDNA stability and the removal of irreparably damaged mtDNA.

TFAM may also increase the buffering capacity of the mitochondrial genome by maintaining mtDNA copy number and limiting the accumulation of functionally defective mtDNA. By supporting mtDNA copy number maintenance and participating in damage recognition or clearance, TFAM may reduce the likelihood that the burden of damaged or mutated mtDNA exceeds the biochemical threshold required to compromise oxidative phosphorylation. This threshold-related role of TFAM should be considered plausible but not yet fully established in aging tissues (Rossignol et al., 2003).

## 4 TFAM in mitochondrial stress, mtDNA-driven inflammation, and cellular senescence

Mitochondrial quality control is essential for maintaining mitochondrial function during aging. Disruption of TFAM homeostasis may impair mitochondrial respiration, increase ROS production, promote mtDNA instability, and enhance mtDNA-driven inflammatory signaling. These processes are particularly relevant to aging because mitochondrial dysfunction, impaired autophagy, cellular senescence, and chronic low-grade inflammation are tightly interconnected.

### 4.1 TFAM, respiratory dysfunction, and ROS imbalance

TFAM supports mitochondrial respiratory function mainly by maintaining mtDNA transcription, mtDNA copy number, and mitochondrial genome stability. Because mtDNA encodes 13 essential subunits of oxidative phosphorylation complexes, TFAM deficiency or dysfunction can impair respiratory-chain assembly and activity. Impaired oxidative phosphorylation reduces ATP production and increases electron leakage from the electron transport chain, particularly at complexes I and III, thereby promoting excessive mitochondrial ROS generation (Murphy, 2009; Zorov et al., 2014).

Under physiological conditions, ROS act as signalling molecules involved in cellular adaptation, differentiation, immune defence, and stress responses (Zhang et al., 2016). However, excessive ROS production during mitochondrial respiratory dysfunction disrupts redox homeostasis and damages lipids, proteins, and nucleic acids (Pizzino et al., 2017). Mitochondrial ROS can directly damage mtDNA, and mtDNA damage can further impair respiratory-chain function, creating a self-amplifying cycle of mitochondrial dysfunction and oxidative stress. A substantial body of evidence has demonstrated that oxidative stress is closely associated with aging and chronic diseases like cardiovascular diseases, renal disease, diabetes, lung disease, and neurological disorders (Valko et al., 2007; Jomova et al., 2023).

Adequate TFAM expression helps preserve mtDNA integrity and respiratory-chain stability, thereby limiting excessive ROS production. In several experimental models, TFAM overexpression or recombinant human TFAM treatment improved mitochondrial respiration, reduced oxidative stress, and alleviated tissue dysfunction, including models of brain aging, myocardial injury, heart failure, and diabetic complications (Ikeuchi et al., 2005; Hayashi et al., 2008; Thomas et al., 2012; Chandrasekaran et al., 2015; Ikeda et al., 2015). These findings suggest that, in certain aging and disease contexts, restoration of TFAM function may have potential therapeutic benefits.

As discussed above, excessive TFAM accumulation relative to mtDNA may reduce mtDNA accessibility, suppress mitochondrial gene expression, and potentially impair respiratory function. Thus, the relationship between TFAM and ROS balance is likely dose dependent and context dependent. In aged cells, mitochondrial proteostasis, autophagy, and mtDNA maintenance are often compromised; therefore, either TFAM insufficiency or an imbalanced TFAM-to-mtDNA ratio may contribute to respiratory dysfunction and ROS accumulation.

### 4.2 TFAM, mtDNA instability, and inflammatory signaling

Mitochondrial DNA instability provides a direct link between mitochondrial dysfunction and inflammation. Damaged, oxidized, or mislocalized mtDNA can act as a DAMP. Because of its bacterial origin and relative enrichment in unmethylated CpG motifs, mtDNA can be recognized by innate immune sensors when it is released from mitochondria into the cytosol, endosomes, extracellular space, or circulation.

TFAM is closely related to this process because it regulates mtDNA stability and nucleoid integrity. When TFAM function is impaired, mtDNA may become depleted, damaged, or abnormally packaged. Respiratory dysfunction and ROS accumulation can further oxidize mtDNA and increase its immunostimulatory potential. Increased mitochondrial membrane permeability, defective mitophagy, mitochondrial permeability transition, or impaired lysosomal degradation may allow mtDNA or TFAM-bound mtDNA complexes to escape from mitochondria.

Once released into the cytosol, mtDNA can activate the cyclic GMP-AMP synthase (cGAS)-stimulator of interferon genes (STING) pathway to induce interferon responses and a multitude of inflammatory cytokines. It can also be recognized by Toll-like receptor 9 (TLR9), primarily within endosomal compartments, which also triggers inflammation (Riley and Tait, 2020; Hu and Shu, 2023). Furthermore, oxidatively damaged mtDNA is not only an activator of the aforementioned pathways but also a potent direct activator of the NOD-like receptor family, pyrin domain containing 3 (NLRP3) inflammasome, driving the maturation and release of potent pro-inflammatory cytokines and even triggering pyroptosis (Shimada et al., 2012; Ning et al., 2025). In aged tissues, persistent mtDNA release or inefficient mtDNA clearance may contribute to chronic sterile inflammation.

When mtDNA is released from damaged mitochondria, TFAM can remain bound to mtDNA. Recent evidence indicates that TFAM contains an LC3-interacting region that enables it to interact with LC3B and target TFAM-mtDNA complexes to the autolysosomal pathway. Through this mechanism, TFAM can act as an autophagy receptor for cytosolic mtDNA, promoting degradation of immunostimulatory mtDNA and limiting inflammatory signaling (Liu et al., 2024).

Therefore, TFAM may influence mtDNA-driven inflammation in two complementary but context-dependent ways. On the one hand, TFAM helps maintain mtDNA stability within mitochondria and may reduce the release of damaged mtDNA by preserving mitochondrial genome integrity. On the other hand, TFAM may facilitate the timely autophagic degradation of leaked mtDNA. However, if autophagy is impaired or TFAM-mtDNA complexes are released extracellularly, TFAM-bound mtDNA may still contribute to DAMP signaling. Thus, TFAM-mediated inflammatory regulation depends not only on its ability to protect intramitochondrial mtDNA but also on the efficiency of downstream mtDNA clearance pathways.

### 4.3 TFAM, mtDNA instability, cGAS–STING-mediated SASP, and cellular senescence

The above mechanisms provide a plausible link between TFAM dysregulation, mtDNA instability, and cellular senescence. Disruption of TFAM homeostasis may compromise mtDNA packaging, transcription, copy number maintenance, and genome stability, thereby promoting mtDNA instability and mitochondrial respiratory dysfunction. Impaired oxidative phosphorylation can increase mitochondrial ROS production, while excessive ROS may further damage mtDNA and reinforce a feed-forward cycle of mitochondrial stress (Correia-Melo and Passos, 2015). Under conditions of impaired mitochondrial membrane integrity, defective mitophagy, or insufficient lysosomal degradation, damaged or TFAM-bound mtDNA nucleoids may accumulate in the cytosol. Cytosolic mtDNA can activate the cGAS–STING pathway and thereby induce inflammatory cytokines and interferon-response genes. This mtDNA–cGAS–STING axis may contribute to senescence-associated secretory phenotype (SASP) amplification and the maintenance of senescence-associated inflammatory states (Martini and Passos, 2023).

Experimental studies provide mechanistic support for this connection. In mice with T cell-specific *Tfam* deletion, mitochondrial dysfunction in T cells promotes inflammatory cytokine accumulation, systemic senescence, and premature aging-like phenotypes, supporting a broader link between TFAM-dependent mitochondrial failure, inflammaging, and senescence (Desdín-Micó et al., 2020). In senescent cells, cytosolic mtDNA has been shown to co-localize with TFAM, suggesting the presence of TFAM-bound mtDNA nucleoids outside mitochondria (Liu et al., 2024). These structures may serve as substrates for cGAS and thereby contribute to cGAS–STING-dependent SASP activation. Consistently, Tfam^+/−^ mouse embryonic fibroblasts reach replicative senescence earlier than wild-type cells and show increased senescence-associated β-galactosidase (SA-β-gal) activity, *Cdkn2a* expression, NF-κB-dependent inflammatory cytokines such as interleukin-6(IL-6), and type I interferon-response genes such as *Ifit3* (Victorelli et al., 2023). Importantly, STING inhibition suppresses pro-inflammatory factor expression without preventing senescence-associated growth arrest or classical senescence markers, suggesting that TFAM-related mtDNA stress may preferentially amplify the inflammatory/SASP component of senescence rather than independently initiate the full senescence program (Victorelli et al., 2023).

## 5 Regulation of TFAM in aging

TFAM abundance and activity are regulated at multiple levels, including transcriptional control, post-transcriptional regulation, mitochondrial import, post-translational modification, and mitochondrial proteolytic turnover. These regulatory mechanisms are particularly important in aging because nutrient sensing, mitochondrial biogenesis, protein import, proteostasis, and autophagy are often altered in aged cells.

### 5.1 Transcriptional regulation of TFAM and aging-related changes

TFAM is encoded by the nuclear genome, and its transcription is coordinated with nuclear programs that regulate mitochondrial biogenesis. The proximal promoter of the TFAM gene contains binding sites for nuclear respiratory factors, particularly NRF1 and NRF2, which link TFAM expression to broader nuclear control of mitochondrial gene expression and oxidative phosphorylation capacity (Virbasius and Scarpulla, 1994; Gugneja et al., 1996). A major upstream regulator of this pathway is peroxisome proliferator-activated receptor gamma coactivator-1alpha (PGC-1alpha), a transcriptional coactivator that promotes mitochondrial biogenesis by coactivating NRF1/2 and other transcription factors. Activation of the PGC-1alpha-NRF1/2 axis can increase TFAM transcription, thereby supporting mtDNA maintenance and mitochondrial gene expression (Qian et al., 2024). This pathway provides an important link between cellular energy demand and mitochondrial genome homeostasis.

TFAM can therefore be positioned as a downstream effector node of aging-related nutrient- and energy-sensing pathways. During aging, reduced NAD^+^ availability, decreased SIRT1 activity, and impaired AMPK responsiveness may weaken PGC-1alpha activation and thereby reduce NRF1/2-TFAM transcriptional output in some tissues (Jäger et al., 2007; Reznick et al., 2007; Cantó et al., 2009; Wang et al., 2015; Marin et al., 2017; Fan et al., 2021). Through these mechanisms, dysregulated SIRT1, AMPK, and PGC-1α signaling may alter TFAM expression and indirectly affect TFAM availability within mitochondria, TFAM-to-mtDNA stoichiometry, and mitochondrial nucleoid organization.

TFAM expression can also be regulated by additional transcriptional and post-transcriptional mechanisms. For example, p53 has been reported to influence mitochondrial gene expression and to interact functionally with TFAM under some stress- or exercise-related conditions (Saleem and Hood, 2013). Several microRNAs also regulate TFAM expression. miR-23b can directly downregulate TFAM in glioma cells, whereas TFAM overexpression in this context may promote tumor cell proliferation (Jiang et al., 2013). miR-155 can suppress TFAM expression and reduce mitochondrial biogenesis, whereas inhibition of miR-494 can increase TFAM expression and mtDNA content in skeletal muscle cells (Yamamoto et al., 2012; Quiñones-Lombraña and Blanco, 2015). These findings suggest that transcriptional and post-transcriptional regulation of TFAM is highly context dependent and may differ across tissues, stress conditions, and disease states.

### 5.2 Post-translational modification and mitochondrial import

After synthesis in the cytoplasm, TFAM must be imported into mitochondria to perform its canonical functions. Immature TFAM contains an N-terminal mitochondrial targeting sequence that directs its translocation into the mitochondrial matrix, where this sequence is cleaved to generate mature TFAM. TFAM import is critical because even when TFAM transcription is normal or increased, impaired mitochondrial delivery can reduce the availability of functional TFAM in the mitochondrial matrix. In diabetic or high-glucose conditions, TFAM gene expression may increase while mitochondrial TFAM protein levels, mtDNA copy number, and mitochondrial gene expression decrease. This discrepancy has been attributed, at least in part, to post-translational ubiquitination and degradation of TFAM during or before mitochondrial import (Santos and Kowluru, 2011; Santos et al., 2014).

Post-translational modifications further regulate TFAM stability, DNA binding, and degradation. Phosphorylation of the HMG1 domain of TFAM by cAMP-dependent protein kinase reduces its DNA-binding capacity and promotes LONP1-mediated degradation (Lu et al., 2013). In *Drosophila*, a mitochondrial cAMP phosphodiesterase stabilizes TFAM by limiting cAMP/PKA signaling in the mitochondrial matrix, thereby promoting mtDNA replication (Zhang et al., 2015). These findings suggest that compartmentalized cAMP/PKA signaling can rapidly tune TFAM function by altering TFAM-mtDNA binding and proteolytic susceptibility.

Other post-translational mechanisms may also affect TFAM. Prolyl hydroxylation has also been implicated in TFAM regulation. Hydroxylated TFAM can bind the von Hippel-Lindau tumor suppressor protein, reducing TFAM phosphorylation and LONP1-mediated degradation and thereby extending TFAM half-life (Li et al., 2022). LDHB has been reported to interact with LONP1 and partially inhibit LONP1-mediated TFAM degradation, whereas HMOX1 can compete with LONP1 for LDHB binding, thereby enhancing TFAM degradation in foam macrophages and contributing to mitochondrial dysfunction during atherosclerosis progression (Peng et al., 2025). These findings indicate that TFAM activity is regulated not only by expression level but also by modification-dependent import, binding, and degradation.

As noted above, TFAM is mainly degraded by LONP1, an ATP-dependent mitochondrial matrix protease that maintains mitochondrial proteostasis by degrading misfolded, unassembled, and oxidatively damaged proteins. LONP1 is especially relevant in aging because chronic oxidative stress and cellular senescence are often accompanied by impaired mitochondrial proteostasis and altered stress responses. In addition to general protein quality control, LONP1 directly contributes to mtDNA homeostasis by regulating TFAM turnover. Selective degradation of free or mtDNA-unbound TFAM by LONP1 helps maintain the TFAM-to-mtDNA ratio and may prevent nucleoid hypercompaction and mitochondrial transcriptional repression. Experimental LONP1 suppression can promote excessive TFAM accumulation on mtDNA and impair mitochondrial transcription. However, whether and how aging-associated changes in LONP1 activity directly alter intramitochondrial TFAM abundance remains incompletely defined and requires tissue-specific investigation.

## 6 TFAM in systemic aging and age-related diseases

### 6.1 TFAM in neurodegenerative, cardiovascular, and metabolic diseases

TFAM dysregulation has been associated with several age-related diseases, particularly in high-energy-demand tissues such as the brain, heart, skeletal muscle, and kidney. These tissues depend heavily on mitochondrial oxidative phosphorylation and are therefore vulnerable to mtDNA instability and respiratory-chain dysfunction.

In neurodegenerative diseases, impaired mitochondrial biogenesis and mtDNA maintenance are common pathological features. In Alzheimer’s disease, downregulation of the PGC-1alpha-NRF1-TFAM pathway has been observed in the hippocampus, suggesting reduced mitochondrial biogenesis and mtDNA maintenance capacity (Sheng et al., 2012). In Parkinson’s disease, reduced TFAM levels, mtDNA depletion, and respiratory-chain deficiency have been reported in substantia nigra neurons, suggesting that TFAM insufficiency may be associated with dopaminergic neuronal vulnerability (Grünewald et al., 2016). In experimental models of brain aging, recombinant human TFAM treatment improved mitochondrial respiration, reduced oxidative stress, and enhanced learning and memory, suggesting that restoration of TFAM-related mitochondrial function may have neuroprotective potential (Thomas et al., 2012).

In cardiovascular aging and heart disease, TFAM appears to be closely linked to mtDNA preservation and mitochondrial respiratory capacity. In a mouse model of myocardial infarction, TFAM overexpression attenuated mtDNA copy number loss, improved respiratory-chain activity, and reduced cardiac dysfunction (Ikeuchi et al., 2005). Similarly, overexpression of TFAM or TWINKLE increased mtDNA copy number, reduced mitochondrial oxidative stress, and improved cardiac remodeling in a heart failure model (Ikeda et al., 2015). These findings suggest that TFAM may be protective in cardiac tissue when mitochondrial dysfunction is driven by mtDNA depletion or oxidative stress. Whether TFAM enhancement is beneficial across all types or stages of cardiovascular aging remains uncertain.

In metabolic disease, TFAM has been linked to insulin resistance, diabetes-associated mitochondrial dysfunction, and diabetic complications. In experimental diabetic neuropathy, TFAM regulation is associated with mitochondrial degeneration and neuronal dysfunction, whereas TFAM restoration shows protective effects (Chandrasekaran et al., 2015). In skeletal muscle, TFAM overexpression enhances fatty acid oxidation and attenuates high-fat diet-induced insulin resistance, potentially through improved mitochondrial function and altered metabolic signaling (Koh et al., 2019). TFAM has also been implicated in diabetic kidney disease, where mitochondrial dysfunction, oxidative stress, and mtDNA instability are central pathological features (Yu et al., 2024).

However, evidence linking TFAM to age-related diseases should be interpreted cautiously. In neurodegenerative, cardiovascular, and metabolic disease models, TFAM changes may reflect primary pathogenic mechanisms, secondary consequences of mitochondrial damage, or compensatory responses. Moreover, acute injury, heart failure, hyperglycemia, lipid overload, and insulin resistance do not fully recapitulate the gradual and multifactorial nature of physiological aging. Because most current evidence comes from disease tissues or experimental models rather than longitudinal studies of normal aging, the relevance of TFAM modulation to aging-targeted therapy requires further validation.

### 6.2 TFAM and systemic aging

Experimental evidence supports a link between TFAM-dependent mitochondrial dysfunction and aging-like inflammatory phenotypes. For example, T cell-specific Tfam deletion in mice causes mitochondrial respiratory dysfunction in CD4^+^ T cells and induces systemic features resembling accelerated aging, including kyphosis, reduced subcutaneous fat, decreased muscle strength, impaired cardiac function, and reduced locomotor activity, together with increased inflammatory cytokine production (Desdín-Micó et al., 2020).

In prematurely aging mtDNA mutator mice, TFAM modulation also produces highly tissue-specific outcomes. In the liver and colon, TFAM overexpression did not meaningfully increase mtDNA copy number or rescue mitochondrial dysfunction; instead, it was associated with reduced mtDNA gene expression and aggravated OXPHOS defects. In contrast, TFAM overexpression increased mtDNA copy number in the spleen and improved cytokine abnormalities, and previous work showed that it could rescue early-onset male infertility in the testis. Conversely, reducing TFAM levels improved brown adipose tissue homeostasis and restored thermogenesis-related markers such as uncoupling protein 1 (UCP1) (Kremer et al., 2025).

Together, these observations indicate that TFAM is not uniformly protective but acts in a tissue- and context-dependent manner in aging-related mitochondrial dysfunction. Accordingly, therapeutic strategies should aim to restore TFAM homeostasis rather than simply increase TFAM abundance. However, direct evidence that TFAM alone initiates cellular senescence or systemic aging across most tissues remains limited. TFAM expression, mtDNA copy number, and circulating mtDNA levels may vary by tissue, disease state, and compensatory mitochondrial biogenesis. Its value as a biomarker or therapeutic target requires tissue-specific and longitudinal validation.

## 7 Therapeutic targeting of TFAM in aging: limitations and future directions

Although current evidence suggests that TFAM has important roles in mtDNA stability, mitochondrial transcription, mitochondrial biogenesis, and aging-related mitochondrial dysfunction, much of the evidence still comes from cell and animal models. Direct evidence from human aging tissues, clinical samples, and longitudinal cohorts remains relatively limited. In addition, existing animal studies are not yet sufficient to fully reflect the dynamic changes and long-term effects of TFAM during natural aging, because many studies are limited to specific genetic backgrounds, single tissues, short observation periods, or artificial overexpression/knockdown models.

Therefore, the age-related dynamics of TFAM in humans, its tissue-specific roles, and its causal relationship with the progression of age-related diseases require further validation. The expression level of TFAM, the TFAM-to-mtDNA ratio, mtDNA copy number, and their effects on mitochondrial function may differ substantially among tissues. High-energy-demand tissues, rapidly renewing tissues, and relatively low-proliferative tissues may not tolerate or adapt to TFAM alterations in the same way. Accordingly, a protective effect observed in one tissue should not be simply extrapolated to other tissues.

Another important issue is that the therapeutic window for targeting TFAM remains undefined. Insufficient TFAM may lead to mtDNA copy number reduction, nucleoid instability, and mitochondrial dysfunction. However, excessive TFAM may also disrupt the TFAM-to-mtDNA ratio, promote mtDNA overpackaging, and suppress mitochondrial transcription or replication. Therefore, simply increasing TFAM is unlikely to produce sustained benefit in all settings.

Particular caution is needed in proliferative or cancer-prone contexts. Under some pathological conditions, TFAM-supported mitochondrial biogenesis and mtDNA maintenance may provide metabolic advantages to highly proliferative cells. In cancer-prone tissues, premalignant lesions, or cells with pre-existing abnormal proliferative signaling, long-term TFAM enhancement could potentially support mitochondrial adaptation, energy supply, and cell survival, thereby increasing tumor-related risks. This possibility remains context dependent and requires direct experimental validation, but it should be considered when proposing TFAM-centered anti-aging interventions.

## 8 Conclusion

**FIGURE 1:**
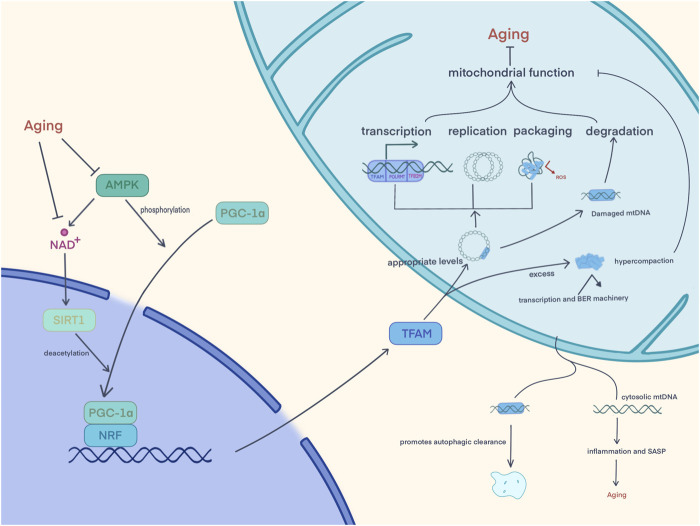
Proposed model of aging-related AMPK/SIRT1/PGC-1α dysregulation, TFAM homeostasis, mtDNA maintenance, and inflammatory signaling. Aging may disrupt energy- and nutrient-sensing pathways by impairing AMPK responsiveness, reducing NAD^+^ availability, and weakening SIRT1 activity. These changes may limit AMPK-mediated phosphorylation and SIRT1-mediated deacetylation of PGC-1α, thereby reducing PGC-1α/NRF1/2-dependent transcriptional regulation of TFAM in some tissues. As a major mtDNA-binding protein, TFAM contributes to mitochondrial genome maintenance by regulating mtDNA transcription, replication, packaging, and turnover. Balanced TFAM levels support mtDNA organization and mitochondrial function, whereas excessive TFAM or an increased TFAM:mtDNA ratio may induce mtDNA hypercompaction and reduce the accessibility of mtDNA to transcription and BER machinery. Aging-related mitochondrial dysfunction and oxidative stress may further promote mtDNA damage, mtDNA leakage into the cytosol, and activation of inflammatory and SASP-related responses. TFAM may also participate in limiting cytosolic mtDNA accumulation by promoting its autophagic clearance. Together, this model highlights the context-dependent role of TFAM in linking aging-related metabolic signaling, mtDNA homeostasis, mitochondrial dysfunction, and inflammation.

TFAM is a central regulator of mitochondrial genome maintenance, linking mtDNA packaging, transcription, replication support, copy number control, and genome stability. By acting directly at the level of mtDNA homeostasis, TFAM occupies a relatively downstream position in mitochondrial regulatory networks and may connect upstream aging-related stress pathways with mitochondrial dysfunction, oxidative stress, mtDNA release, innate immune activation, impaired quality control, and cellular senescence. Current evidence suggests that altered TFAM expression or activity is associated with multiple aging-related phenotypes and age-related diseases, supporting its potential relevance as both a mechanistic mediator and a therapeutic target (Figure 1).

However, TFAM should be viewed as a homeostatic regulator rather than a uniformly protective factor. Both TFAM deficiency and excessive or dysregulated TFAM accumulation may impair mitochondrial genome maintenance and mitochondrial function, depending on TFAM-to-mtDNA stoichiometry, tissue context, metabolic state, and protein quality-control capacity. Therefore, increasing TFAM abundance alone may not necessarily improve mitochondrial function and may even be maladaptive in specific tissues or disease states. Future studies should clarify whether TFAM alterations during aging are causal events, compensatory responses, or secondary consequences of mitochondrial stress. Tissue-specific and longitudinal human studies, together with physiologically relevant aging models, will be essential for defining the therapeutic window of TFAM-centered interventions. Overall, future therapeutic strategies should aim to restore TFAM homeostasis rather than indiscriminately increase TFAM expression.
